# Wrapper Functions for Integrating Mathematical Models into Digital Twin Event Processing

**DOI:** 10.3390/s22207964

**Published:** 2022-10-19

**Authors:** Reiner Jedermann, Walter Lang

**Affiliations:** IMSAS—Institute for Microsensors, -Actuators and -Systems, University of Bremen, 28359 Bremen, Germany

**Keywords:** digital twins, event processing, wrapper functions, real-time models, cool chain, Apache Kafka, intelligent container

## Abstract

Analog sensors often require complex mathematical models for data analysis. Digital twins (DTs) provide platforms to display sensor data in real time but still lack generic solutions regarding how mathematical models and algorithms can be integrated. Based on previous tests for monitoring and predicting banana fruit quality along the cool chain, we demonstrate how a system of multiple models can be converted into a DT. Our new approach provides a set of generic “wrapper functions”, which largely simplify model integration. The wrappers connect the in- and outputs of models to the streaming platform and, thus, require only minor changes to the model software. Different scenarios for model linking structures are considered, including simultaneous processing of multiple models, sequential processing of life-cycle-specific models, and predictive models, based on data from the current and previous life cycles. The wrapper functions can be easily adapted to host models or microservices from various applications fields, to predict the future system behavior and to test what-if scenarios.

## 1. Introduction

Digital twins (DTs) represent physical assets in the virtual word. The virtual representation is constantly updated by sensor information from the physical object. DTs provide a solid, standard, and scalable abstraction layer [1]. The application can interact with the asset without being aware of the hardware implementation of the sensor system. DTs go far beyond merely representing sensor data; rather, they provide conclusions from the data, predictions of future system behavior and of non-directly measurable quantities. According to [2], “a digital twin is a virtual, dynamic model in the virtual world that is fully consistent with its corresponding physical entity in the real world and can simulate its physical counterpart’s characteristics, behavior, life, and performance in a timely fashion”.

Such analytic capabilities [3] require integration of multiple models originating from different disciplines. Depending on the application, the list of contributors reaches from operation research and material science to process, chemical, and biological engineering.

The main question of this article is to find a method for easy integration of such models into a DT software platform as separate software entities with a focus on models that directly process sensor data. The goal of our research is the development of a set of generic ‘wrapper functions’ that require only minor code extensions to host different models.

### 1.1. Software Technologies for Modelling in Digital Twins

The review article on the modelling perspective of DTs [4] lists 550 related papers including applications in health care, meteorology, manufacturing and process technology, education, and the transportation and energy sector. Such a detailed review is far beyond the scope of this article. In the following study, we summarize the major required software technologies for model integration in DTs.

In the past, the necessary set of models has often been integrated into a single, coupled monolithic unit [3], but lacking scalability and flexibility. They suggest an approach where every part of the analytic process is embedded in a microservice.

**Microservices** are a software pattern to create complex applications from individual processes that are loosely coupled. The application can include microservices written in different languages [5]. They can be developed, deployed, and changed independently from one another [3]. Models that originate from different disciplines and contributors remain in separate software units, which is a more natural method for their implementation.

Stateless behavior, in which the microservice must not retain a session state from previous requests, is preferred for microservices by [6], making it easy to duplicate, suspend, or move the service to other computational resources. However, most process models are time-dependent and require information from the past system state. To solve this contradiction, a solution to store states in a separate data stream was developed in [6]. Other authors do not prescribe a stateless behavior; instead, ref. [7] recommends integrating an individual database to each microservice.

The **even-driven architecture** is a common method to connect microservices asynchronously [7]. Refs. [8,9] recommend this architecture for the implementation of DTs. Sensors and models publish their measurements and results to a streaming platform. These messages are considered as events. All information is organized by topics. Subsequent models and consumers subscribe to certain topics to be notified on each new available event in the stream.

The strongest characteristics of this architecture are listed as performance, scalability, fault tolerance, and evolutionary by [7], making the architecture very suitable for monitoring a large set of objects by a DT platform. Evolutionary means that new features can be added easily, at best without interrupting the whole system.

Besides various commercial solutions [4], Apache Kafka is a widely used open-source **streaming platform** for event processing, as in [6,8,10]. Kafka was designed for high-performance applications. If the number of objects to trace increases, the platform can be scaled up by including additional brokers on separate servers and by running the event processors for the models in the network instead of on a single server. If one processor instance fails, the remainder of the networks continues operation. Software libraries for programming publish-and-subscribe interfaces are available for programming languages such as Python and Java. Matlab required special handling by programming an intermediate Java interface [11]. The performance tests in [5] showed that a Kafka cluster with three brokers running on different virtual machines can handle 8000 messages per second of size 256 Bytes equivalent to 2 Mbyte/s of data.

The message queuing telemetry transport (**MQTT**) protocol is widely used for integration of sensors into IoT solutions. The Eclipse MQTT Mosquitto broker or the Hono protocol adapter was combined with the Kafka streaming platform to a DT in several further projects, e.g., [3]. Some authors further enhanced the DT by including a database for long-term storage and access by query languages. MongoDB was used in [5] and InfluxDB in [9].

The human-readable JavaScript Object Notation (**JSON**) data format is used in almost all listed Kafka-based DT applications. Binary formats, such as Protobuf, can reduce the message size by a factor of six [12] at the cost of losing flexibility. After adding fields to the message structure, a protocol header must be recompiled and distributed in the system.

### 1.2. Processing of Analog Sensor Data

Several DT solutions are found in logistics and manufacturing applications, e.g., [13]. Related models have often been limited to material flow on a shop floor or a logistic transport network. The models entail discrete states and process discrete sensor data, such as the position of a work piece at a particular machine.

However, a detailed digital representation of the work piece should also consider its internal states, such as mechanical wearing, remaining lifetime, and the effects of deviating transport and environmental conditions. These properties require analog sensors, e.g., for temperature and analytic models to process these analog or continuous data. More examples for ‘analog’ DTs can be found in the chemical processing industry [14] and in agriculture [15].

Several commercial DT applications are based on artificial intelligence and machine learning [16]. The automated learning process requires tremendous amounts of training data. The training data must contain sufficient instances of all combinations of input factors. Otherwise, the machine learning overfits to single deviating events [17]. Physical modelling, e.g., computational fluid dynamics [15], are less demanding in regard to the size of training data.

Models must be updateable [18,19], i.e., they incrementally process the sensor data every time a new measurement is published. The analytics often entail estimation of quantities than cannot be directly measured, so-called ‘hidden’ states [20]. Such state observers [19] are widely used in fabric automation to estimate and noise-filter the location of robots, e.g., [21], in the chemical industry [14] and for bioreactors [22,23].

Often, the entire modelling of all physical equations is too complex for real-time processing in DTs. The authors of [24] recommend surrogate models in this case for mapping model input to output data by regression or interpolation methods. They have found wide application in the chemical industry [14].

Models are mostly written in different software frameworks by people from different disciples using different interfaces. **Wrapper functions** are used as mediator or format translator, as in [10]. In their specific example solution, they use a wrapper to connect the Kafka streaming platform with the simulation via an Excel file. A commonly agreed definition of wrapper functions is still lacking in scientific literature. Some definitions can be found in company bulletins and discussion forums:

According to [25], “wrappers are used for two primary purposes: to convert data to a compatible format or to hide the complexity of the underlying entity using abstraction”.

Wrappers can encapsulate code blocks of another programming language. They can act as proxy by transforming a local method call to a network message for running the implementation on another machine. They can compose multiple underlying implementations in a single call. They can enforce ‘separation of concerns’. The user should not know the details of the implementation, and the implementation should not have to care for code on the user side, e.g., for data display [26].

Models for continuous inputs signals play an important role for DTs in most application fields. This is especially the case for DTs based on analog sensors, e.g., for temperature, humidity, and airflow in a cooling chamber. Hard real-time constraints are often not necessary; therefore, we prefer the term ‘live’ sensor data to ‘real-time’. In summary, a DT for processing analog live sensor data consists of mathematical models marked by the following features:

- The models predict non-measurable internal properties of the object, i.e., values for which no sensor is available or cannot be installed in a specific location.
- They conclude system behavior from sensor measurements and outputs of preceding models. For example, the reduction in product lifetime or a gradual loss of product quality depending on the sensed deviations from the optimal storage and handling conditions.
- They also predict future system behavior.
- Models can originate from different disciplines; they can be presented in multiple mathematical forms and programming languages.
- Their algorithms can range from a simple integral for calculating accumulated quality loss up to a complex set of differential equations or any other form.

### 1.3. Concept for Model Integration

The approach to integrate different sub-models as separate services into a DT platform has been hindered by the effort required for manually writing the necessary interfaces one by one. We address this problem by our new concept to simplify the integration of models by providing a set of generic wrapper functions to connect various types of models with a Kafka streaming platform. Our purpose is to separate the programming of the models from the programming of access functions to the DT platform. The first task can be best performed by scientists and experts from the related fields, whereas the latter task falls in the area of information technology.

This is achieved by wrapper functions, which read all necessary inputs from the DT platform, call the model, and after the model has completed computation, they write back the model results to the DT platform. The task of assigning the correct input and output streams and time windows in a system with a vast number of physical objects is shifted from the models to a wrapper function.

If the models are linked in a linear chain or ‘pipeline’ structure, with each model having exactly one predecessor and one successor, linking is straightforward. In many applications, the relations between models are more complex. Often, models require multiple inputs, such as sensor data, the current output of other models, and collected data from earlier life-cycles phases; examples are discussed in Section 2.

Recent solutions such as [10] relied on individual programming of wrappers. As added values we provide a set of standard wrapper functions covering models with different types or combinations of inputs. The adaption of a suitable wrapper to a specific model requires only minor extensions of an inherited class. This concept enables us to easily plug in new models to the streaming platform or exchange them with a newer version. We like to show that splitting the model in services does not create significant computation time overhead.

On the software side, wrappers are programmed as classes holding different adapter methods; however, we prefer the keep the common term “wrapper functions”.

### 1.4. Method of Implementation

In this paper, we describe the steps that were necessary to develop our new concept of generic wrapper functions. A set of models for the monitoring of cool chain processes was developed in our earlier research [17], including models for temperature-related quality changes, analysis, and prediction of temperature changes over time, and estimation of heat production of fruits during ripening as ‘hidden’ or not-directly-measurable state.

Possible linking patterns of these models were analyzed in Section 2. Beside a simple pipeline structure, three other patterns were identified.

We developed a concept of how the identified patters can be represented in software by a chain of consecutive topics. This novel concept of so-called ‘enriched streams’ is presented in Section 3. Section 4 introduces a concept to describe the linking structures by a configuration file.

Our final concept for wrapper functions is presented in Section 5. The wrappers connect to the streaming platform in the same way as microservices. However, a stateless design was not feasible. Most models describe a time-dependent behavior and, thus, must store information from the previous iteration cycle. Extracting these states from the model code would require major changes inside the models, which originate from different contributors. An instance of the wrapper with local states is created for each freight item or work piece and runs in a separate thread. Therefore, they can be better described as virtual objects [3] than complying with a strict concept of microservices.

In Section 6, we show the wrapper concept in action for our example application on the refrigerated ocean transport and subsequent artificial ripening of bananas. As a pre-condition, we assume that the models already have a software interface in form of an update method to calculate the new model prediction for the next time interval with the current sensor values as input arguments.

The results in Section 7 are evaluated regarding the created overhead. The concept to route all communication between the models through the streaming platform requires time for network access and, thus, increases the latency compared to a monolithic model that would only require a single access to the streaming platform. As latency, we define the time span between publishing the sensor data and reading of the final model result. Performance tests of our wrapper solution were carried out by playing back recorded sensor data at accelerated speed. The sensor data from 10 freight items were fed into the DT platform in parallel at a speed of 10 messages per second each. In total, 100 values per second were sent to the platform. All models were executed on an i7 workstation. Furthermore, the size of additional code to adapt each model to the generic wrapper is evaluated in the discussion in Section 8. Section 9 closes with conclusions.

## 2. Examples for Linked Model Structures

The complex behavior of a physical or biological object can be best analyzed when different properties are described by separate models. The set of models can be linked according to various typical patterns. By analyzing the model relations in our example application, we identified four typical cases. Models can be executed quasi-simultaneously during the same life-cycle phase of the object, or in sequence in different phases. Models can be arranged in a linear chain or form a meshed network.

### 2.1. CASE 1: Simultaneous Chain Processing

**Figure 1:**
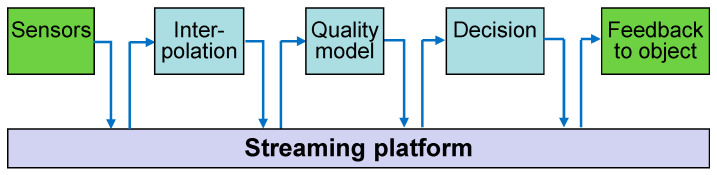
Linear chain/pipeline of three models and connection to the real world via publishing and subscription to a streaming platform.

In our first scenario, these models are updated simultaneously, i.e., after each new sensor measurement, all models in the chain are activated. Data are forwarded through this model pipeline (Figure 1). For example:

The first model converts the sensor data, e.g., to calculate absolute from relative humidity, to interpolate available sensors for positions in between or to deduce the object’s core temperature by measurements on its surface.

The second model calculates the resulting quality loss.

The third process implements the decision algorithms, e.g., by correcting the cooling setpoint if a quality problem is foreseen.

Models were programmed by different experts; a direct linking of the models would require the programming of complex proprietary interfaces. Instead, the models are linked through a common streaming platform, which enables the model interfaces to be programmed independently of their later use and of their linking with other model types, as described in Section 3.

### 2.2. Case 2: Life Cycle Dependent Models

Unfortunately, the real scenario is often more multifaceted than the above-described linear chain of models, particularly if different models must be applied in various life-cycle phases of the object.

We have worked for several years on a remote-sensing and quality-prediction system for refrigerated ocean containers with fruits [17]. We are currently attempting to enhance the system by taking the most advantage of new DT concepts and features. Therein, the combination of models for the live processing of sensor data turned out to be one of the crucial challenges. So far, the linking of models has only been tested in offline simulations.

### 2.3. Example Application for Remote Cool Chain Monitoring

We have already developed a set of models to predict the behavior of bananas along the cool chain from farm to processing in Europe. The focus of this paper is on wrappers for the in- and outputs of the models, their possible linking, and with less emphasis on their inner structure. Mathematical details of model implementation can be found in our previous publications [20,27].

Although our tests focused on the international delivery chain, the approach can be extended to the final steps of the cool chain, i.e., delivery to distribution centers and local retail stores. If a constant fruit quality is maintained by sensor monitoring, modeling, and intelligent stock rotation, waste is reduced.

**Figure 2:**
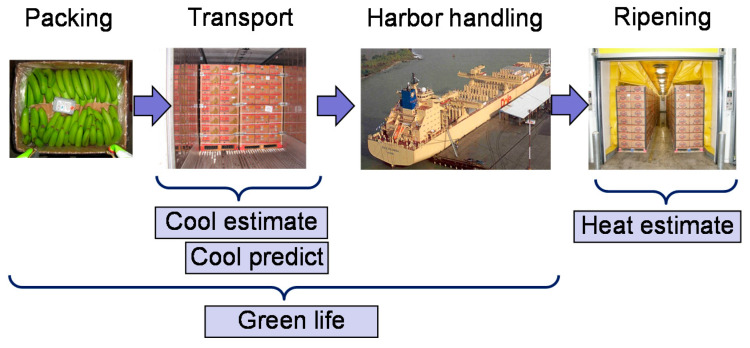
Banana chain and valid timeslots for models.

In our project, we considered the first four steps (Figure 2) of the banana chain. *1. Packing:* The bananas are harvested, washed, and packed into boxes in Central America. *2. Transport:* The bananas are loaded “warm” to a refrigerated container and cooled down during ocean transport. The supply air setpoint is typically set to 13.0–14.4 °C. *3. Harbor Handling:* Cooling is interrupted during harbor handling in Europe. *4. Ripening:* By exposure to the gas ethylene, an artificial ripening process is initiated, which converts starch to sugar and thereby generates large amounts of heat.

During the project, we developed four models, which are already available in an updateable format to process sensor events:

1. The green life model predicts the remaining number of days until an unwanted ripening process starts, and the bananas can no longer be used for commercial processing. This model takes the box temperature *T_Box_* over time as the input.
2. A parameter for cooling efficiency *k_M_* can be estimated from the measured box *T_Box_* and supply air *T_Supply_* temperatures during the cooling phase.
3. After 3 days, a stable estimate for *k_M_* is available. The same model can then be used for prediction of the future box temperature, typically under the assumption that the supply air temperature remains at a constant value.
4. The amount of heat generated during ripening is an indicator of the progress of this process. The last model to estimate the ripening heat requires the *k_M_* as a single value and *T_Box_* as a time series.

All models are only valid during specific phases of the banana chain (Figure 2). Model execution must be activated and stopped by transport events, such as “start of transport” or “completion of ripening”.

**Figure 3:**
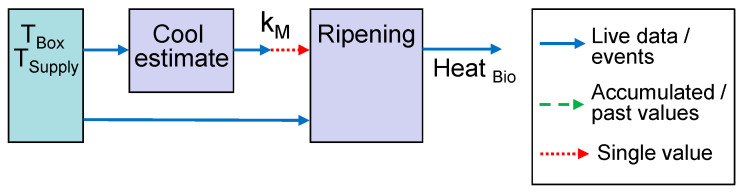
Model linking for estimation of ripening heat based on parameter *k_M_* for cooling efficiency.

In our first example for life-cycle-dependent models, the first model estimates the parameter *k_M_* during the transport and cooling phase. The second model for ripening heat is only active after the first model has completed. Both models require a subset or time window of the temperature sensors *T_Box_* and *T_Supply_*. The sensor readings are passed as events, whereas for *k_M_*, only a single finally estimated value is required (Figure 3).

### 2.4. Case 3: Predictive Models

**Figure 4:**
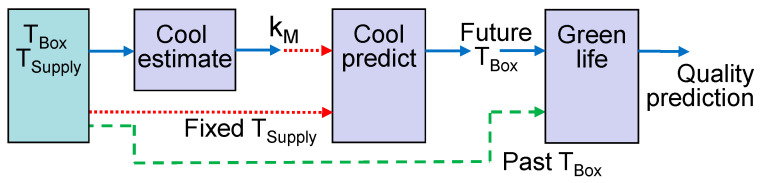
Prediction of future temperature and green life after operator query.

Models cannot only be applied to analyze the current state of the physical object, but also to predict its future. For the latter case, model linking through data streams has to be rearranged. For example, the operator can query the predicted temperature *T_Box_* upon arrival of the container in Europe. Queries can be initiated 3 or 4 days after transport begins when sufficient sensor data for model estimation are available (Figure 4). The first model is the same as in the example in Figure 3, except for the fact that the interim estimate of *k_M_* is read at an earlier time point. If the query is repeated a second time, a later and probably more accurate value for *k_M_* will be read. The second model predicts the future development of *T_Box_* by taking the current *T_Box_* as the initial state and the last known value of *T_Supply_* as the constant input. Finally, the green life model combines the accumulated data for *T_Box_* from the time window before the query was initiated with the future prediction for T_Box_.

The latter example can be extended to a what-if scenario. Rather than assuming that the setpoint remains unchanged, the operator can test the effect of interventions by changing the setpoint. In an advanced DT solution, a further processing unit would test different possible interventions and automatically apply the most beneficial one to the real object.

### 2.5. Case 4: Meshed Model Neworks

The sensor information can be processed in two parallel paths, and joined together in a final step. Such a complex scenario was not required to model our banana chain. Nevertheless, it is easy to construct a fictive example: Temperature data can be evaluated by two separate models, the first for fruit firmness and the second to predict changes of fruit color. A final model combines their outputs to a united quality index. Such a scenario can create synchronization issues. If one of the first two models takes more time for processing, events can become out of sync.

## 3. Enriched Streams

The provision of streaming platforms for model integration and linking is one of the new key features of DTs. In our approach, we follow an architecture suggested in [9]. The use of three different database solutions entails some redundancy but offers high flexibility for data access. Sensors use the standard MQTT (message queuing telemetry transport) protocol to publish their measurements to Eclipse Mosquitto as protocol adapter. Access to data in table form and by standard queries is possible via the InfluxDB database. All components and the models are linked through the Kafka streaming platform as the central broker [11,27].

The integration of the models into a streaming platform is straightforward for a consecutive chain of models, as in case 1. For the more complex cases, information has to travel over parallel paths, if the structures in Figure 3 and Figure 4 are directly translated to a set of publish and subscribe interfaces.

**Figure 5:**
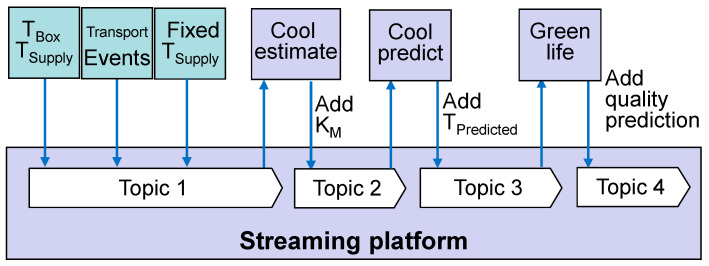
Enriched data stream consisting of successive topics with additional information.

However, programming of models is largely simplified when models have to listen to a single topic only. We therefore suggest a new approach to link models by a consecutive chain of topics. We call this approach “enriched streams”. Each model copies all available information from an input topic to an output topic but enriches the stream with its estimation and prediction results. For example, the predictor scenario of Figure 4 can be rearranged to a linear stream of subsequent topics, as shown in Figure 5. Sensor data, transport events, and commands to initiate the prediction at a certain point of time are written to the first topic. The cool-estimate model adds the identified parameter for cooling efficiency *k_M_* to the stream. The cool prediction adds an array with the future estimated temperature values to a single message, according to the point of time, when the prediction was triggered. Finally, the green life model combines the past temperature values from the stream with the array of predicted values and adds the quality prediction.

Blocking and synchronization problems, due to reading data from different streams, can be mostly avoided by this approach, although it entails some data overhead because topics must be copied multiple times.

Often, it is also possible to rearrange a meshed model linking structure to a linear chain. For the fictive example in case 4, it is not necessary to execute the two quality models strictly in parallel. Instead, the firmness model can first process temperature data and add its prediction to the stream, followed by the color model. The final evaluation model reads both data from the same stream.

## 4. Formal Description of Model Structure

Next step for converting the model chain into a DT is the conversion of the graphical linking structure to a formal description, i.e., a configuration file. The configuration file contains linking information for each model. Box 1 shows the configuration of one of the required models for the cargo type “Bananas” as an example.

The ripening model subscribes to the output of the identification of the cooling parameters (CoolPara). The estimated ripening heat is published to a topic with the suffix “RipePara”.

The configuration file can contain individual model linking schemas for different cargo types. When is new shipment is announced, the cargo type is selected, and a specific configuration is generated by replacing the term “’#ID” with the container or shipment number.

The file also contains a translation from the transport events to specific commands for the models. For example, at the event “Start_Transport”, the ripening model starts to collect estimated values from the cooling identification. The update process for model itself is only started after the “Start_Ripening” event is received.

The specific configuration is published to a general “Model_Config” topic, to which handlers for all available model types subscribe. If they detect their own model name in the data stream, they initiate a new model instance.

Box 1 **Code snippet 1: Configuration example for the ripening model.**

**Bananas:**

     **…**

     **RipeningModel**:

 **Topic_In**: Transports_Shipment_#ID_CoolPara

 **Topic_Out**: Transports_Shipment_#ID_RipePara

 **LifeCycleTranslate**:

 **…**

 **Start_Transport**: Start_Collect

 **Arrive_Transport**: Stop_Collect

 **Start_Ripening**: Start_Model

         …

## 5. Wrapper Functions

The goal of the wrapper functions is to separate the programming of the mathematical model from the access to streaming platform and model management.

We assume that the model algorithm is already available in an updateable form, i.e., it can process sensor data and other types of input information directly after each measurement interval. Predicted and estimated parameters are updated in each interval, preferably in real time. The model algorithm can be kept unchanged. The only requirement is that it provides some kind of *step()* function to calculate one model update.

Making models updateable requires mathematical and programming effort. A general method for this conversion is not available, although several mathematical methods can be used, such as parameter estimation techniques and state space observers, namely the Kalman filter [19]. However, the connection of the updateable model to the streaming platform can be largely simplified by the suggested use of wrapper functions.

### 5.1. Collector Wrapper Example

Most of the models can be described by a collector structure. They collect information during earlier life-cycle phases without updating their model output during this period. In a later phase, the model makes use of the collected data to update its prediction for each new measurement. For example, the ripening model collects the *k_M_* estimates during the transport phase. Model updates are calculated based on the last *k_M_* value during the subsequent active phase, after the “Start_Ripening” event is received.

The collector phase can be omitted in case that no input from earlier life-cycle phases is required. The model of the chain in Figure 1 can be integrated to the streaming platform by the same wrapper structure.

### 5.2. Wrapper Software Structure

**Figure 6:**
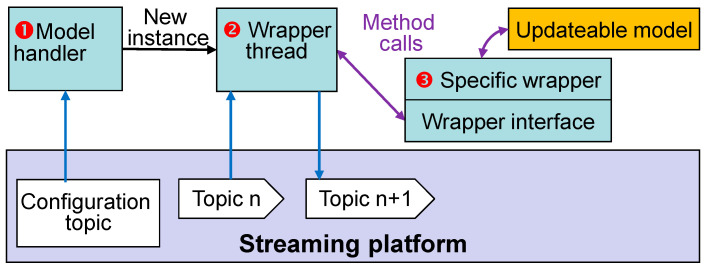
General wrapper software structure.

The general structure of wrappers consists of three software entities (Figure 6):

1. The model handler manages multiple transport items. The handler subscribes to the configuration topic and starts a new thread, i.e., a model instance for each item.
2. The second entity contains only generic methods. This wrapper thread provides general functionality for the models, such as subscribing and publishing to the streaming platform and mapping of transport events to state transitions of the model, e.g., from waiting state to the collector phase and the active phase. Model execution is started and stopped accordingly.
3. The model-specific part for each model type translates the model outputs to a JSON structure and vice versa for the model inputs. The specific wrapper is called through a wrapper interface, which defines abstract *step()* and *collect()* methods.

### 5.3. Specific Wrappers

All access to the streaming platform is performed by the model handler and the wrapper threads, which are independent of the individual model types.

Only the specific wrappers must be written anew for each model type. Transferring the required in- and outputs from JSON to function arguments for calling the mathematical model must be performed individually. Each model requires a different number of in- and outputs with different descriptive names. In order to avoid model-specific programming in the first two entities, in- and output values are forwarded as a JSON structure and only read out and written back in the specific wrapper. Except for JSON, no special libraries or programming techniques are required in this entity.

Besides the JSON translation, data preprocessing and model-specific tasks are also implemented in this level, such as verifying if the sampling interval is inside the allowed range or filling missing sensor values with the last known one.

For some models, such as the cooling parameter identification, the calculation of the output value must be postponed until sufficient input samples are available.

### 5.4. Wrappers for Prediction Models

The prediction models in Figure 4 and Figure 5 require some extension to the collector wrapper. The user can publish a “query” event to the sensor data topic. The wrapper has to process this event and start the related model algorithm through an additional *prediction()* function in the wrapper interface. The prediction result consists of an array of consecutive model outputs, e.g., the predicted temperature in intervals of 1 h until the estimated arrival of the transport. The prediction array is added to a single message and published to the model’s output topic.

## 6. Demonstration and Testing

Two scenarios were evaluated in detail as proof of concept and for testing their functionality of the DT platform. The wrapper function and models for the ripening scenario (Figure 3) and the prediction of future temperature and green life (Figure 4) were programmed in Java. An alternate implementation of the green life model and its wrapper was written in Python to demonstrate the platform’s capability to integrate models in different programming languages.

**Figure 7:**
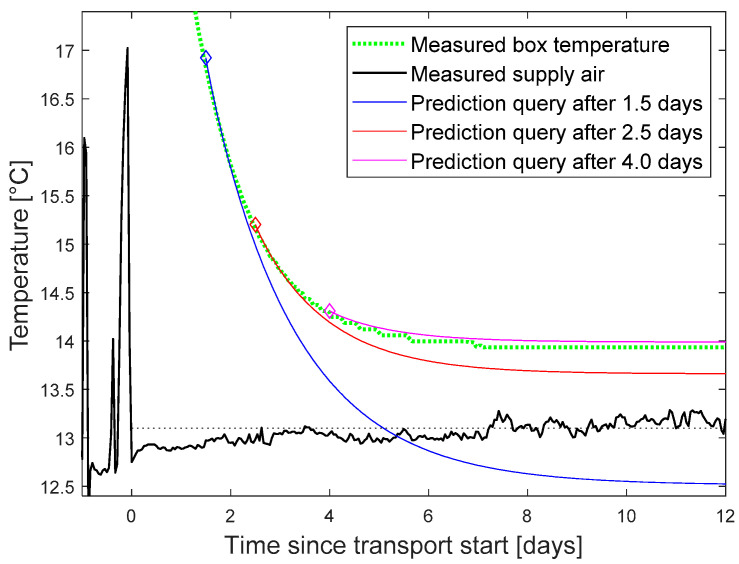
Measured and predicted temperature. Prediction for assumed constant supply air temperature of 13.2 °C.

In the following, we focus on the temperature and green life prediction as the more complex scenario. A prediction query was triggered at different points of time after transport start. The setpoint was assumed to remain at the average of the last known values of 13.2 °C. The predicted curve for the box temperature was compared with the finally measured curve as reference (Figure 7).

**Figure 8:**
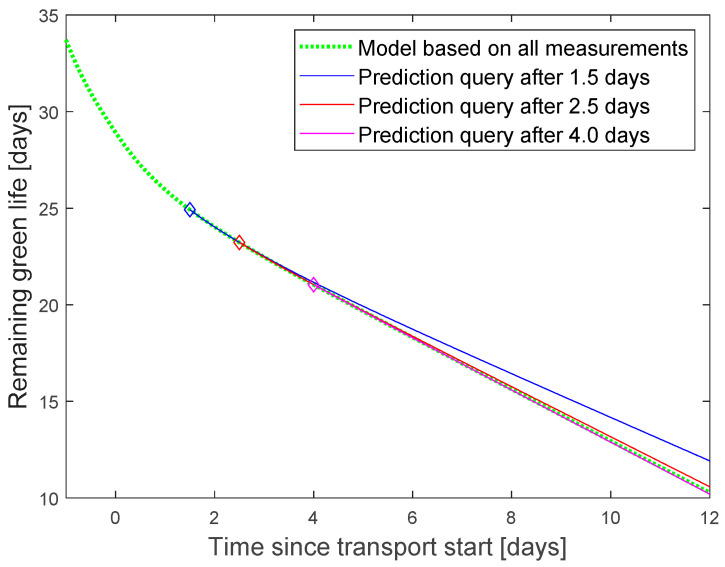
Green life prediction based on full temperature curve (dotted green) and predicted temperature for different query times. Supply air temperature assumed constant at 13.2 °C after the query.

The reference for the green life was calculated by using the finally measured temperature curve as input to the model (Figure 8 dotted green line).

Prediction queries were inserted into the data stream between 1.5 and 4 days after transport start. They use the model structure in Figure 4 to estimate the green life, based on the predicted temperature. The green life prediction error was calculated as the difference between predicted value and reference model for the point of time, when the ship arrives the harbor. Additionally, a what-if scenario was tested to predict the effect of adjusted setpoint and cooling air temperature.

Our test scenario is based on real-life data, which we collected during earlier experiments for the sea transportation of bananas and their further processing in Europe [17]. It was more useful to play back these data for scenario testing rather than using new real-world live data by repeated tests. For the streaming platform, it does not matter from where data are coming, as long as they use the same MQTT interface and have the same sampling interval. The play back speed can be even higher, e.g., measurements that were taken in intervals of 1 h are played back with a speed of 10 per second for up to 10 banana boxes in parallel. The performance of the platform was measured in terms of latency between sending the sensors values and availability of the final model result. The tests were carried out with the streaming platform running on a virtual Linux server with four cores, all models were running on a local Windows i7 workstation. Timing measurements were based on the *System.currentTimeMillis()* function provided by the operating system.

## 7. Results

Our focus was to demonstrate the platform and verify its performance. Additionally, the accuracy of the green life prediction was tested.

### 7.1. Platform Performance

The tests proved the functionality of our DT platform and of the wrapper functions. Prediction results were displayed in the graphical interface of InfluxDB. For better layout, the final graphics were plotted in Matlab (Figure 7 and Figure 8).

The Kafka platform was able to handle the model linking with only minor latency. For the first test, only a single freight object was monitored by the DT platform. The delay from sensor reading to the last model in the chain was measured to 3.5 ms in average including four publish/subscribe interactions with the streaming platform. The latency increased to 60 ms during a load test for the simultaneous monitoring of 10 objects, which was still less than the sampling interval of 100 ms.

In contrast to Kafka, InfluxDB showed performance issues in our tests. Sending the results from 100 models per second to InfluxDB caused data congestion with an average delay of 20 s until all write operations were completed.

### 7.2. What-If Scenario

**Figure 9:**
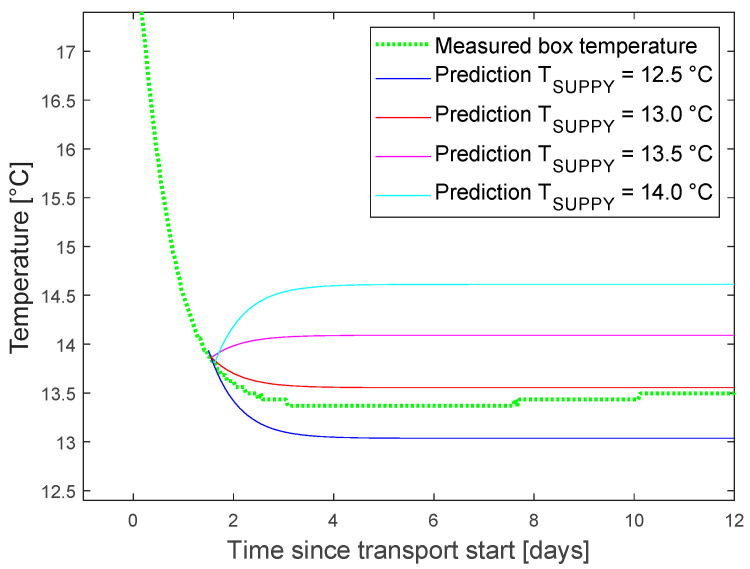
What-if scenario for testing setpoint changes, 1.5 days after transport start. Measured box temperature (green) for supply air with average 13.2 °C, see black line in Figure 7 left.

The output of one what-if scenario is given in Figure 9 as an example. The effects of a change of setpoint, 1.5 days after start of the transport was tested. The setpoint can be slightly increased to save energy or decreased to extend green life. Approximately one day of green life is gained by a setpoint reduction of 0.5 °C.

### 7.3. Prediction Accuracy

**Figure 10:**
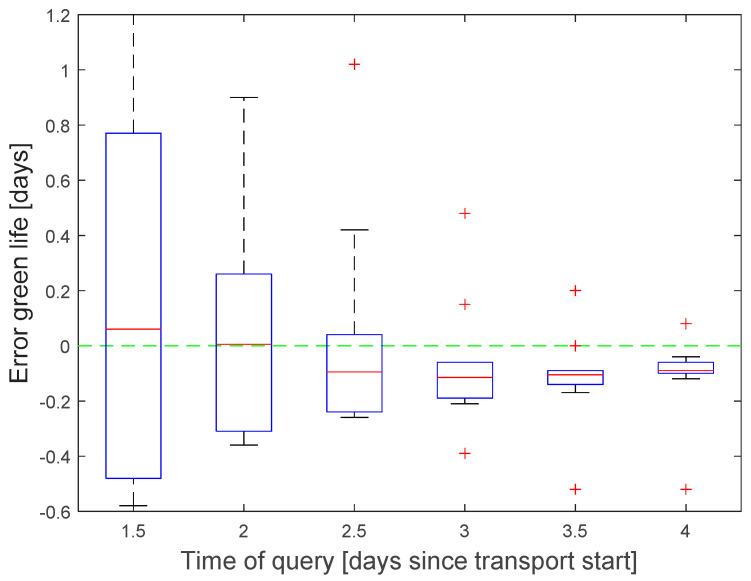
Boxplot of green life prediction error as function of query point of time.

The accuracy of the green life prediction increased with the amount of collected data before the query was initiated. Three days after transport start, the typical error falls below 0.2 days with some outliers up to 0.5 days (Figure 10). Lager outliers for earlier queries are lying outside the plotting range (2.2 day for query after 2 days, 5.9 for 1.5 days).

The error of the temperature prediction was typically below 0.2 °C, 3 days after transport start, with outliers up to 0.4 °C.

### 7.4. Code Size

The programming overhead for integration sub-models instead of a monolithic solution to the DT platform was evaluated based on the code size of the required specific wrapper functions. For example, the wrapper for the ‘Cool-Ident’ model required in total 46 lines of code. Six lines of constructor code initialize internal variables and the model. The method to process sensor events implements the *step()* function from the interface. It needs 12 lines of code for reading temperature values from JSON, verifying the time interval via a shared class, calling the model internal update method and testing if sufficient data are available to carry out the parameter estimation. The consecutively called method to write back the estimated model parameter to the output topic requires six lines.

## 8. Discussion

This section starts with practical limitations, which are specific to our use-case but not to the wrapper concept in general. Our concept is based on the event-driven architecture, whose pros and cons will be summarized before the final evaluation of additionally required CPU time and coding overhead for our solution.

### 8.1. Practical Limitation in Our Use-Case

Prediction of future temperature and its effect on green life is only possible after an accurate estimation of the cooling model parameters has become available. Otherwise, the prediction is distorted by larger outliers. A reliable prediction is only possible after most of the temperature difference is already compensated. A more detailed discussion of model accuracy and means for improvement is beyond the focus of this article on software architecture.

The second limitation concerns the application of the what-if scenario for setpoint changes. In practice, there is very little freedom to adjust the setpoint. At lower temperature, bananas are harmed by so-called chilling injuries. A more realistic application would include the prioritization of containers with low expected green life in the harbor. A what-if scenario should test the effect on other containers, which must be postponed in turn. The DT is extended to a system of systems [28], with the twin for box quality integrated into a twin for harbor management.

### 8.2. Event-Driven Architecture and Microservices

The event-driven architecture is very suitable for monitoring a large set of objects by a DT platform due to its high performance, scalability, and evolutionary features [7]. However, as every other software pattern, the advantages have to be balanced against some drawbacks. Although single event processors can be tested easily, testing is difficult in a large, meshed model structure. Timing becomes critical if events are forwarded on parallel paths, and the output of the last processor depends on the sequence of incoming events. The publisher does not receive feedback whether the event is correctly processed by the subsequent subscribers, making error handling difficult. A tool to keep trace of the event flow must be programmed separately.

The microservice pattern offers large flexibility in programming individual software units. New models and software version can be started any time. In our solution, only the configuration file must be edited to link the new model with other existing ones.

### 8.3. Computational Costs

The streaming platform proved to have a sufficient low latency for most applications, even with a sub-optimal setup as in our tests. The Kafka cluster consisted of only a single broker on a virtual server with one partition. All publish/subscribe interactions of the models with the platform were handled via the campus Ethernet.

With the current solution, ten objects can be traced in intervals of 100 ms. With a total of four model steps, including transfer to and from the MQTT broker, this summed up to 400 interactions per second, causing a delay of 60 ms in average.

If the application requires a shorter update interval, or more objects should be traced, several elements of the platform can be optimized, e.g., increasing the number of brokers on separate servers, divide each broker into partitions, using dedicated servers instead of virtual machines shared with other users, and running models and platform on the same server to avoid delays by Ethernet communication. For example, the Kafka setup in [5] was tested for a maximum throughput of 8000 messages per second with three brokers and 10 partitions each.

All models were programmed in incremental format and required only marginal CPU time per step. Delays by Ethernet communication and of the Kafka broker had a higher contribution to the total latency. An exact quantification of the individual contributions was hardly feasible due to the limited resolution of *the System.currentTimeMillis()* function.

The code of our generic wrappers does not need further changes. Only one specific adapter function must be modified for each sub-model, with typically 50 lines of code to be edited.

Neither the additional computing costs for the sub-model-oriented integration nor the additional programming effort create significant obstacles for applying our concept. Monolithic models can be replaced by a microservice-oriented style.

### 8.4. Further Architecture Features

Every object is handled by a separate chain of topics in our solution. For each object, a new thread instance is started for the required models. This approach makes the programing of wrapper functions straightforward. They do not have to filter data; they just subscribe to the related topic. Each model instance runs independently, runtime software errors only affect a single object. Linux provides 65,000 threads by default; however, in a large cool chain scenario with thousands of sensors, the use of individual threads and topics can cause performance issues or reach limitations of the operating system.

Each model adds new information to the incoming data and publishes the combination to an enriched stream, thus storing redundant data. Due to the availability of hard disks in Terra Byte range and fast Ethernet communication, this point is less crucial.

InfluxDB was included in our architecture for long-term data storage, for providing tabular access by query languages, and as interface to graphical display tools. The performance of InfluxDB will be sufficient for a real-word scenario, with sensor data arriving in intervals of minutes or hours. However, our accelerated testing with 100 write operations per second caused performance issues and data congestions. Therefore, it should be questioned if the same functionality can be achieved without InfluxDB as mediator or another database provides better performance.

## 9. Conclusions

We demonstrated the capability of DTs for enhanced monitoring of industrial processes. Based on our scenario for the cool chain monitoring of bananas, we showed that DTs can represent sensor data, estimate resulting quality changes, predict the future behavior of the object, estimate not-directly-measurable states, such as the heat production during ripening, and evaluate the effects of changes in the process parameters by simulating what-if scenarios.

DTs in Industry 4.0 started with the modelling of material flow and continued with an increasing integration of analog sensors, e.g., for temperature and mechanical stress. The analysis of such analog sensor data needs detailed modelling, which if often too complex for monolithic solutions. Microservices are a common method to combine independent software units into a complex application. The related event-driven architecture has already found its way into DTs and Industry 4.0, but mostly for sensor data collection, offline machine learning, and simulation. We demonstrated how these software architectures can also be applied to integrate multiple sub-models for sensor data processing into DT platforms. Although we focused on an example for a specific cool chain, our approach is not limited to the related sensors or models. The generic wrappers can be adapted to various sensor models in industrial applications.

Our novel concept largely reduces the programming effort for integration of new models. The provided set of generic wrapper functions covers pipeline and meshed model linking structures, including what-if scenarios. The wrappers handle the access to the streaming platform and assignment of model inputs and outputs to suitable topics. The individual programming per model is reduced to changing a few lines of code in an adapter function.

In comparison to monolithic solutions with all sub-models combined in a single software unit, the additional computation cost in terms of CPU time sums up to 1 ms per sub-model, monitored object, and iteration. This additional CPU load is negligible in most sensor application with update intervals of minutes or even hours. The stability of our platform was tested for an interval of 100 ms. If shorter intervals or a higher number of sub-models and objects are required, the performance of the Kafka platform can be increased by separating each broker into partitions increasing the number of brokers on additional servers.

### Future Work

So far, we covered various DT application scenarios by the four different cases for model linking structures (Section 2) with our set of generic wrapper functions. The list of linking structures might be still incomplete. For example, spatial interpolation models to predict values for uncovered positions in between the sensor locations require a high number of sensor inputs. The set of generic wrapper functions should be extended in the future accordingly. Furthermore, the wrapper functions were mostly programmed only in Java, except for a simple example in Python and a general method to connect Matlab models. In future developments, the complete set of wrappers should be translated to other programming languages.

Despite these open tasks, we provided a solution that offers the same flexibility in programming as microservices. New or updated models can be easily plugged into the streaming platform. The novel concept for generic wrapper functions provides the necessary means to program the components of sensor data processing models in separate software units, and, thus, simplifies the integration of such model structures and enables more accurate sensor monitoring of objects in an industrial environment.

## Data Availability

Not applicable.
